# Expansion of myeloid-derived suppressor cells contributes to metabolic osteoarthritis through subchondral bone remodeling

**DOI:** 10.1186/s13075-021-02663-z

**Published:** 2021-11-16

**Authors:** Lixia Zhang, Cameron L. Kirkwood, Jiho Sohn, Ashley Lau, Mary Bayers-Thering, Supinder Kour Bali, Sridhar Rachala, John M. Marzo, Mark J. Anders, Frank Beier, Keith L. Kirkwood

**Affiliations:** 1 Department of Oral Biology, School of Dental Medicine, University at Buffalo, The State University of New York, 645 Biomedical Research Building, 3435 Main St, Buffalo, NY 14214-8006 USA; 2 Department of Medicine, University at Buffalo, Buffalo, NY USA; 3 Department of Orthopaedics, University at Buffalo, Buffalo, NY USA; 4 Department of Physiology and Pharmacology, University of Western Ontario, London, Ontario Canada; 5 Western Bone and Joint Institute, University of Western Ontario, London, Ontario Canada; 6 Department of Head and Neck/Plastic and Reconstructive Surgery, Roswell Park Comprehensive Cancer Center, Buffalo, NY USA

**Keywords:** Osteoarthritis, Myeloid-derived suppressor cells, Osteoclasts, Obesity, Subchondral bone

## Abstract

**Background:**

Osteoarthritis (OA) subsequent to acute joint injury accounts for a significant proportion of all arthropathies. Myeloid-derived suppressor cells (MDSCs) are a heterogeneous population of myeloid progenitor cells classically known for potent immune-suppressive activity; however, MDSCs can also differentiate into osteoclasts. In addition, this population is known to be expanded during metabolic disease. The objective of this study was to determine the role of MDSCs in the context of OA pathophysiology.

**Methods:**

In this study, we examined the differentiation and functional capacity of MDSCs to become osteoclasts in vitro and in vivo using mouse models of OA and in MDSC quantitation in humans with OA pathology relative to obesity status.

**Results:**

We observed that MDSCs are expanded in mice and humans during obesity. MDSCs were expanded in peripheral blood of OA subjects relative to body mass index and in mice fed a high-fat diet (HFD) compared to mice fed a low-fat diet (LFD). In mice, monocytic MDSC (M-MDSC) was expanded in diet-induced obesity (DIO) with a further expansion after destabilization of the medial meniscus (DMM) surgery to induce post-traumatic OA (PTOA) (compared to sham-operated controls). M-MDSCs from DIO mice had a greater capacity to form osteoclasts in culture with increased subchondral bone osteoclast number. In humans, we observed an expansion of M-MDSCs in peripheral blood and synovial fluid of obese subjects compared to lean subjects with OA.

**Conclusion:**

These data suggest that MDSCs are reprogrammed in metabolic disease, with the potential to contribute towards OA progression and severity.

## Introduction

Osteoarthritis (OA) is a highly prevalent and disabling disease that remains a challenge to develop effective therapeutics primarily since there is a limited understanding of the early-stage disease which results in later-stage diagnoses [1, 2]. In OA of the knee, there is a progressive loss of cartilage associated with changes in chondrocyte phenotype, including the activation of a catabolic state. In response to several different stimuli, including inappropriate mechanical loading and catabolic factors within the synovium, chondrocytes modify their phenotype and express a subset of factors, such as cytokines, chemokines, alarmins, DAMPs, and adipokines. All of these mediators act as paracrine factors to initiate a vicious cycle of cellular-induced inflammatory processes, which both promote inflammation in the synovium and participate in cartilage damage [3]. In addition, numerous inflammatory cytokines are increased in joint tissues during the acute post-injury phase, including IL-1β, IL-6, IL-17, and TNFα, and act as primary drivers of synovial inflammation [4]. Evidence that innate and adaptive immune systems play roles in the post-traumatic joint exists, with increases in activated macrophages, CD4^+^ T cells, complement (C3a), and inflammatory cytokines described in injured knee joints [5]. Despite this knowledge, current therapies for OA are restricted and no treatment has been conclusively shown to alter OA disease progression. Thus, a deeper understanding of the immunological dynamics that drive OA disease progression, particularly early-stage disease, is warranted to develop precision-based therapies to arrest progression.

The development of obesity and other metabolic diseases is a complex process involving genetic and environmental interactions that connect metabolism with the immune system. Chronic low-grade systemic inflammation in response to obesity (termed metainflammation) is a consequence of immune dysregulation that results from the continuous exposure to bacterial lipopolysaccharide (LPS) and saturated free fatty acids under hyperglycemic conditions [6]. Metainflammation generated by increased circulating cytokines/adipokines may influence immunity and cellular homeostasis [7]. In addition to the current paradigm of inflammation in OA pathophysiology is the awareness that metabolic disease is associated with OA (termed metabolic osteoarthritis), in part through proinflammatory conditions and oxidative stress [8]. This new appreciation of metainflammation and impact in OA creates new challenges and opportunities, especially in this era of precision medicine.

Myeloid-derived suppressor cells (MDSCs) have been widely described as a heterogeneous population of myeloid progenitor cells and immature myeloid cells with potent immune-suppressive activity [9]. Increased frequency of MDSCs has been commonly reported in the context of cancer immunology; however, MDSCs have also been shown to contribute to chronic and acute inflammatory processes associated with infections, immune responses in sepsis, transplantation, autoimmune diseases, and aging [9]. Hence, MDSCs are frequently detected in different inflammatory-based pathological disorders. Since the first characterization of MDSCs, the cellular origin and nature of these cells have been a subject of debate. MDSCs are very similar to monocytes and granulocytes and share common morphologic features. In mice, CD11b^+^Ly6G^+^Ly6C^low^ phenotype of PMN-MDSC is identical to that of neutrophils, and the CD11b^+^Ly6G^−^Ly6C^high^ phenotype of M-MDSC is the same as inflammatory monocytes. In acute infections, it is proposed that MDSCs may have a beneficial role when the stimulus has been cleared by limiting tissue damage produced for a persistent immune response [9]. In contrast, during chronic inflammation, expansion and activation of MDSCs contribute to immunosuppression and oxidative stress [10, 11]. However, differentiation and function of MDSCs are influenced by the inflammatory microenvironment generated, suggesting a disease-specific function of MDSCs. For example, it has been reported in numerous autoimmune diseases that while MDSCs are increased they cannot suppress disease progression [12–14]. Hence, their contribution to pathological processes goes far beyond immune suppression. To date, no published studies have focused on MDSCs in PTOA disease development.

A number of recent publications have reported that MDSCs can function as osteoclast progenitors in pathological conditions with complications associated with bone destruction [10, 11, 15, 16], including collagen-induced arthritis models [17]. However, no studies have addressed MDSCs in the context of osteoarthritis. Obesity has been shown to enhance MDSC expansion and obesity-related metabolic factors [11, 18], and our group has recently shown that MDSCs are metaboloically regrogrammed during osteoclast differentiation [19]. In particular, altered levels of adipokines contribute to OA development by inducing the expression of proinflammatory factors as well as degradative enzymes, leading to the breakdown of cartilage matrix, inhibition of new cartilage matrix synthesis, and stimulation of subchondral bone remodeling [20]. Herein, we report that MDSCs are expanded in OA during metabolic disease and that MDSCs have an increased capacity of MDSCs to degrade subchondral bone that can contribute towards OA progression.

## Materials and methods

### Animals

The animal protocol used in this study was approved by the Institutional Animal Care and Use Committee at the University at Buffalo. All mice were kept in a controlled temperature and environment under a 12-h light/12-h dark cycle. Male C57BL/6J mice were initially purchased from the Jackson Laboratory (Bar Harbor, ME, USA). Diet-induced obesity (DIO) mice were fed with 45% kcal fat diet (D12451; Research Diets; HFD) starting at 4 weeks of age. The control group was fed with 10% kcal fat diet (D12450H; Research Diets; LFD) with all other micronutrients matched. After mice were fed HDF/LFD diets for 12 weeks, the destabilized medical meniscus (DMM) model to surgically induced OA in the mouse knee joint was performed by transection of the medial meniscotibial ligament as we have shown previously [21–23]. Sham control mice had the same surgery except that the meniscotibial ligament was not transected. The mice were housed in groups of 4–5 and allowed free activity. Mice were euthanized 8 weeks after surgery [24]. Only male mice were used for these studies since there is a clear sex difference with DMM severity between sexes [25, 26]. The ARRIVE Guidelines Checklist was implemented for this study.

### Histopathological analyses

Knee joints were dissected free of the skin or excess muscle, formalin fixed, decalcified and processed, and embedded frontally in paraffin. Five-micromolar sagittal sections were generated using a rotary microtome (Leica) of frontal sections to examine the anterior cruciate ligament [27]. Sections were stained with fast green and Safranin-O and evaluated for cartilage damage and synovial inflammation by two independent assessors. Cartilage damage and histological scoring was based upon the OARSI histopathology initiative [28].

### Osteoclast scoring and the subchondral bone thickness, area, and osteoclast density

Following histologic processing serial frontal sections were stained for osteoclasts using tartrate-resistant acid phosphatase (TRAP) stain and fast green for contrast. Osteoclasts were defined as multinucleated (>3 nuclei) giant cells that stained red and were in contact with the surface of trabecular bone within the subchondral bone area. Osteoclast numbers were calculated by two independent evaluators. Five measurements of the TRAP^+^ cells at 200× magnification were made in the subchondral bone area where the average number of osteoclasts presented. Osteoclast area and subchondral bone thickness were also measured following outlining areas of interest using a Wacom board coupled with ImageJ2 software for image and data analysis [29]. Subchondral bone was histologically graded using an established protocol shown to have a significant relationship with the OARSI grading for cartilage using a scoring system of 0–3 where 0 is normal, 1 = mild, 2 = moderate, and 3 = severe changes for subchondral bone changes [30, 31].

### Mouse MDSC isolation and characterization

Single-cell suspensions from the bone marrow of HFD and LFD mice having DMM-induced PTOA and sham control were isolated. Red blood cells were lysed using a lysis buffer (eBioscience, San Diego, USA) and cells were washed with PBS, filtered through a 70-μM nylon membrane to obtain final cell suspensions (1 × 10^6^ per tube), and then incubated with Fc blocked for 10 min, using 2 μl anti-CD32 (BD Biosciences). The following antibodies were added in staining buffer (eBioscience) for 30 min: CD11b-APC (clone M1/70, # 130-113-231, Miltenyi), Ly6G-PE (clone 1A8,130-102-392), and Ly6C-FITC (clone REA796, #130-111-915, Miltenyi). Data was acquired by the BD flow cytometer (Fortessa, BD Bioscience) with data analysis using FlowJo V10.0.7 software (FlowJo, OR, USA). Cytological examination of M-MDSCs was achieved through cytospin isolation and imaging. Briefly, M-MDSC isolated from the bone marrow of LFD and HFD mice was obtained by MACs sorting (described below) and 100μl of a cell suspension was loaded into a cytospin chamber and spun for 5 min at 500 rpm (Cytospin 2; Shandon). Slides were air dried at room temperature and subsequently stained with the LeukoStat staining kit (Fisher Scientific, Pittsburgh, PA).

### In vitro osteoclastogenesis

Primary cultures of mouse osteoclast precursor cells in the form of bone marrow-derived monocytes (BMM) were obtained from femurs and tibias of HFD/LFD fed mice that were DMM/sham operated as described previously [32]. Red blood cells were lysed using an RBC lysis buffer (eBioscience, San Diego, USA), and cells washed with PBS and filtered through a 70-μM nylon membrane to obtain final cell suspensions. MDSC subpopulations were isolated from BMM using autoMACs pro (Miltenyi) by myeloid-derived suppressor cell isolation kit (# 130-094-538, Miltenyi). Granulocytic MDSCs were isolated through a positive selection for Ly6G using the mouse Ly6G microbeads (Miltenyi). The Ly6G^−^ fraction was used to gate to obtain the monocytic MDSC fraction. The monocytic MDSC cells (2.5 × 10^5^ cells) were seeded into 48-well plates in α-MEM (Gibco, USA), 10% heat-inactivated FCS (Hyclone, USA), supplemented with 10 ng/ml monocyte colony-stimulating factor (M-CSF) for 4 days. Osteoclastogenesis was induced with 25 ng/ml M-CSF and 50 ng/ml RANKL for an additional 4 days. Cells were fixed and stained for TRAP activity using a leukocyte tartrate-resistant acid phosphatase (TRAP) kit (Sigma). Osteoclasts were identified as red-stained cells containing three or more nuclei. The number of osteoclasts was enumerated as described by our group [32–35].

### Human subjects

This study was conducted in accordance with the Helsinki Declaration and approved by the University at Buffalo Institutional Review Board. All subjects gave written informed consent for participation. All patient participants were recruited through a patient cohort database of existing patients diagnosed with OA/PTOA from the Buffalo General and Kaleida Health. OA subjects over 18 years of age were recruited with inclusion criteria that included patient subjects who report no autoimmune disease and no history of any immune-modulating systemic medications (including prednisone, biologics, methotrexate, cyclosporine, retinoids). Patients had at least one femoro-tibial joint with OA for inclusion in the pilot study. Exclusion criteria included HIV disease, pregnancy, immunosuppressant medications, anticoagulants or bleeding orders, bisphosphonates or steroids, and antibiotic therapy within the previous 3 months. Body mass index (*BMI*) was used as a surrogate measure to assess body fat. *BMI* was categorized as non-obese (<29.9 kg/m^2^) or obese (>30.0 kg/m^2^).

### Human MDSC immunophenotyping

Peripheral blood was obtained by venipuncture and synovial fluid was obtained by needle aspiration of the femoro-tibial joint OA patients exhibiting joint effusion. Mononuclear cells were isolated from whole blood by Ficoll-Paque density gradient centrifugation and analyzed within 6 h following blood sampling. The following anti-human antibodies were purchased from Miltenyi Biotec (Germany): CD11b-APC (clone M1/70, # 130-113-793), HLA-DR–PE (clone AC122, #130-113-402), CD14-fluorescein isothiocyanate (FITC) (clone Tuk4, #130-113-146), CD15-APCvio770 (clone VIMC6, #130-104-992), CD33-viobright515 (clone REA775, #130-111-027), CD66b-PEvio770 (cloneREA306, #130-119-808), and their corresponding isotype controls, along with corresponding isotype controls. Cellular phenotype was characterized by flow cytometry (Fortessa, BD Bioscience), and data were analyzed with the FlowJo V10.0.7 (FlowJo, OR, USA). Gating was performed according to standard protocols where cellular debris and dead cells were initially gated out. HLADR^−^ and CD11b^+^ populations were selected and then M-MDSC (HLADR^−^CD11b^+^CD14^+^) and PMN-MDSC (HLADR^−^CD11b^+^CD15^+^) obtained from the HLADR^−^ CD11b^+^ populations.

### Statistical considerations

All statistical analysis was performed using GraphPad Prism version 8.2.1 for macOS (GraphPad Software, Inc., San Diego, CA). A two-tailed unpaired *t*-test was used when comparing the means of two variables. A two-way analysis of variance (ANOVA) followed by Tukey’s multiple comparison posttest was used to compare means of greater than 2 variables. Results are presented using an uncertainty with a 95% confidence interval.

## Results

### HFD promotes DMM-induced OA disease pathology

**Fig. 1:**
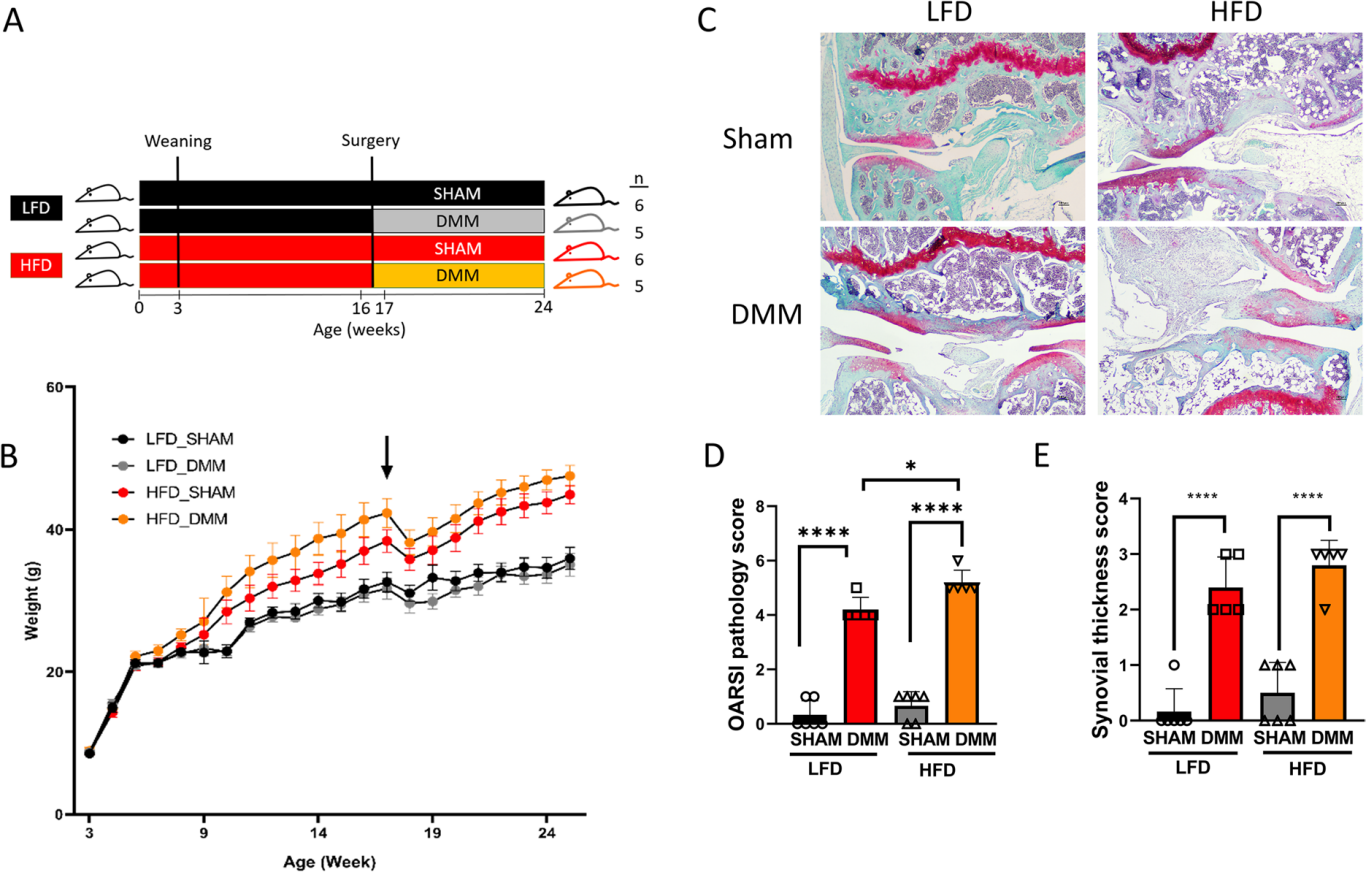
High-fat diet promotes post-traumatic osteoarthritis in mice. **A** Overview of the experimental design. Mice were fed either a low-fat diet (LFD) or a high-fat diet (HFD) for 12 weeks and sham or destabilization of the medial meniscus (DMM) surgery was performed at weeks 16–17. Sham group (*n* = 6), DMM group (*n* = 5). **B** Changes in weight over time. Arrow represents the time of the surgery. HFD increases body weight compared to LFD. **C** Representative histopathological section from mouse knee joints stained with fast green and Safranin-O. **D** Glasson Modified OARSI pathology score measuring osteoarthritic damage (*n* = 6 in the sham group, *n* = 5 in the DMM group). **E** Synovial thickness score (*n* = 6 in the sham group, *n* = 5 in the DMM group). Scale bar = 100μM. **P < 0.01, ****P < 0.0001. Two-way ANOVA Tukey’s multiple comparison test

To investigate the effect of HFD on cartilage, we used the PTOA model induced by surgical destabilization of the medial meniscus (DMM) after mice were fed an HFD or micronutrient matched LFD for 12 weeks. Knee joint tissues from the four groups were harvested 8 weeks after DMM or sham control surgery (Fig. 1A). Mice on HFD weighed significantly more than mice on LFD, while the type of surgery did not affect weight significantly on either diet (Fig. 1B). Histological sections were stained with Safranin-O (glycosaminoglycans) and fast green (bone and tendon). Representative images from each experimental group are presented (Fig. 1C). We performed Glasson Modified OARSI semi-quantitative scoring system to quantitatively assess the pathological difference in experimental groups (Fig. 1D). Qualitatively, the surface of the cartilage was smooth in the sham control group; however, the DMM group showed massive proteoglycan loss. The cartilage in the DMM group presented with denudation and deformation. OARSI scoring data indicated a significant degeneration of articular cartilage in DMM groups compared to the sham control, with significantly higher OARSI scores in HFD/DMM mice than in LFD/DMM mice. These changes were characterized by increased thickness of the synovial membrane layer and disorganized structure.

### HFD increases MDSC expansion and osteoclast differentiation in DMM mice

**Fig. 2:**
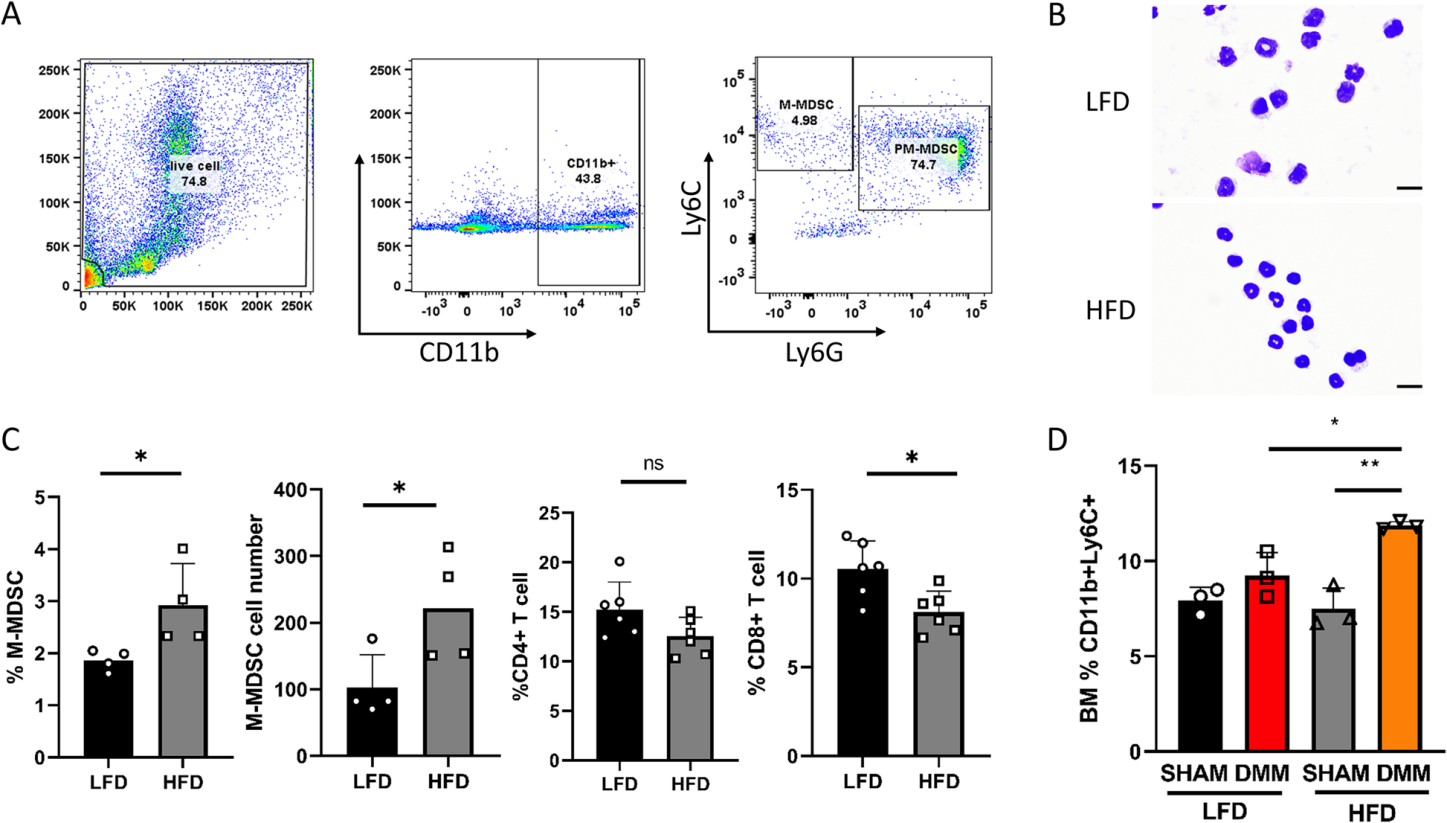
High-fat diet increases monocytic-MDSC expansion during PTOA. **A** Gating strategy used to define the M-MDSC subpopulation (CD11b^+^Ly6C^+^Ly6G^−^ cells) in mouse bone marrow. Following the initial FS/SC discrimination, the gate was set on CD11b^+^ cells. After exclusion of doublets (not shown), live CD11b^+^ cells were gated and Ly6C^+^ and Ly6G^+^ populations. **B** Cytospin images of M-MDSC cells from the bone marrow from LFD and HFD mice (at 16 weeks). Scale bar denotes 10 microns. **C** HFD increases the BM M-MDSC population in mice (*n* = 4 in each group) by total number and percentage with concomitant suppression of CD8^+^ and CD4^+^ T-cells compared to LFD mice (*n* = 6 in each group, at 16 weeks). **D** HFD increases the BM M-MDSC population in DMM mice (*n* = 3 in each group). *P < 0.05, **P < 0.01, ***P < 0.001, ****P < 0.0001 by two-way ANOVA Tukey’s multiple comparison test

**Fig. 3:**
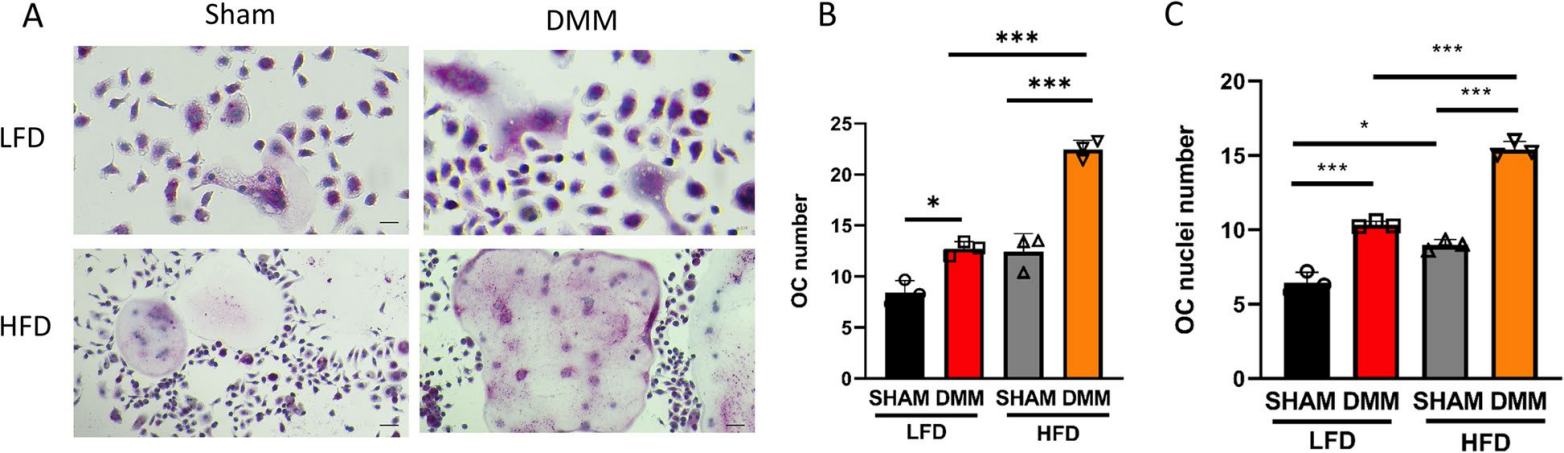
High-fat diet (HFD) increases osteoclastogenesis in DMM mice. **A** Representative images of isolated bone marrow M-MDSC post-M-CSF and RANKL stimulation stained with tartrate-resistant acid phosphatase (TRAP; *n* = 3 in each group). **B** Osteoclast number (*n* = 3 in each group), number of osteoclasts per field of view, each enumeration utilized 5 fields of view/slide at 20×. **C** Osteoclast nuclei number per osteoclast (*n* = 3 in each group). *P < 0.05, **P < 0.01, ***P < 0.001, ****P < 0.0001 by two-way ANOVA Tukey’s multiple comparison test

M-MDSCs are immature myeloid cells classically described as possessing immunosuppressive functions. However, we and others have shown that this lineage can also differentiate into osteoclasts in the presence of macrophage colony-stimulating factor (M-CSF) and receptor activator of NF-kB ligand (RANKL) [10, 15]. To determine the effects of HFD on MDSC levels and the ability to form osteoclasts from HFD/LFD mice that had DMM or sham control surgery, we obtained M-MDSCs from the bone marrow of these mice. Data in Fig. 2A displays the gating strategy used in the present study to obtain M-MDSC (CD11b^+^Ly6C^+^) cells from the bone marrow. To further characterize this population, we used cytospin morphology (Fig. 2B) and flow cytometry from the bone marrow of LFD and HFD mice at 16 weeks of age to determine phenotypic differences (Fig. 2C). The appearance of M-MDSCs from cytospin samples from LFD and HFD appears similar without any obvious differences in cellular morphology. M-MDSCs were expanded (both total number and percentage) under HFD conditions with a concurrent significant reduction of CD8^+^ T-cells and a slight reduction in CD4^+^ T-cells. Figure 2D shows the total number and percentage of M-MDSCs (Ly6C^+^CD11b^+^ cells) in the bone marrow from all four experimental groups of mice, respectively. We observed that M-MDSC cell populations were significantly expanded in the bone marrow after DMM surgery, compared to sham controls. Remarkably, there were significantly more M-MDSCs in the HFD/DMM than in the LFD/DMM mice. Following cell sorting, bone marrow-derived M-MDSC cells were plated with M-CSF and RANKL to induce M-MDSC cellular differentiation into large multinucleated osteoclasts in vitro (Fig. 3A). Quantitative analysis of TRAP^+^ staining of osteoclast formation indicated that the number of osteoclasts in the DMM group was significantly higher than in sham groups, as well as being also higher in HFD than LFD mice (Fig. 3B). M-MDSC cells from the DMM groups exhibited a significantly increased capacity to form mature osteoclasts (more nuclei OC) compared to the sham control (Fig. 3C) with significantly more mature osteoclasts formed in HFD DMM cultures compared to LFD DMM cultures under the same experimental conditions.

### HFD alters subchondral bone parameters

**Fig. 4:**
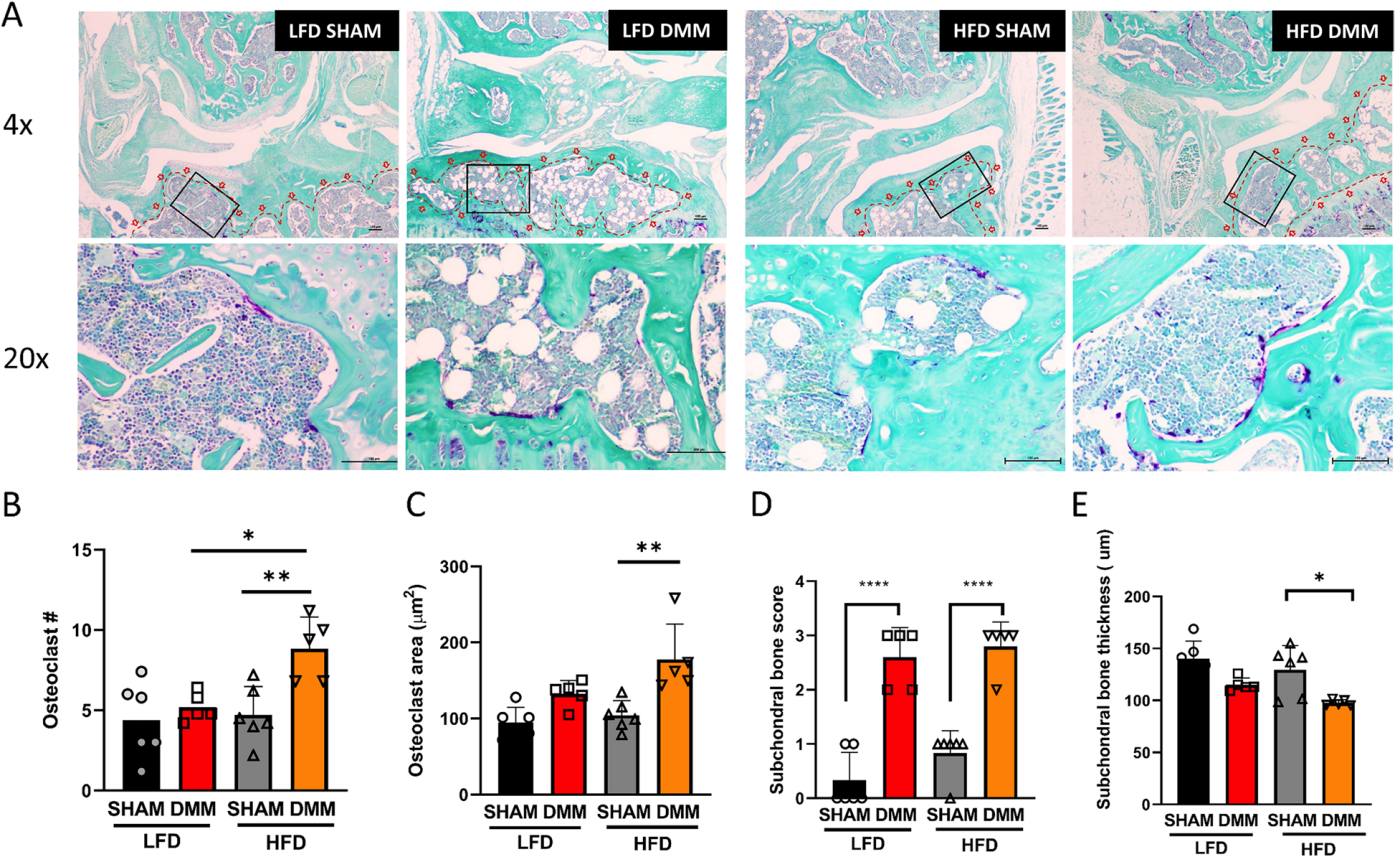
High-fat diet (HFD) alters subchondral bone parameters with increased osteoclast formation. **A** Representative histopathological section from mouse subchondral bone stained with tartrate-resistant acid phosphatase (TRAP). **B** HFD increases osteoclast number in DMM. **C** Osteoclast area (μm^2^). **D** Subchondral bone score. **E** Subchondral bone thickness. *n* = 6 in the sham group, *n* = 5 in the DMM group; scale bar = 100μM. *P < 0.05, ****P < 0.0001 two-way ANOVA Tukey’s multiple comparison test

To understand if in vitro osteoclastogenesis observations translated into exacerbated DMM-induced pathology during the metabolic stress of obesity, we examined the subchondral bone for pathological changes in DMM mice on HFD versus LFD controls. Tartrate-resistant acid phosphatase (TRAP) staining of frontal sections of experimental groups was performed to evaluate the degree of osteoclast formation in the subchondral bone (Fig. 4A). The number of TRAP-positive cells in subchondral bone was significantly increased in DMM/HFD mice compared to sham/HFD or DMM/LFD mice (Fig. 4B). Also, the osteoclast area was significantly increased in DMM/HFD mice compared to sham/HFD. However, we did not observe any significant differences with the osteoclast area within the subchondral bone of the tibia or femur sections in all other comparisons, including DMM versus sham in LFD or in DMM in HFD versus LFD (Fig. 4C). Still, we discovered that the subchondral pathology score increased in both DMM groups, but without a significant difference between LFD or HFD DMM experimental groups (Fig. 4D). Lastly, there was a reduction in subchondral bone thickness in DMM/HFD compared with sham/HFD, and a trend towards subchondral bone thickness reduction in the DMM groups (Fig. 4E).

### MDSCs are expanded in obese patients with OA

**Fig. 5:**
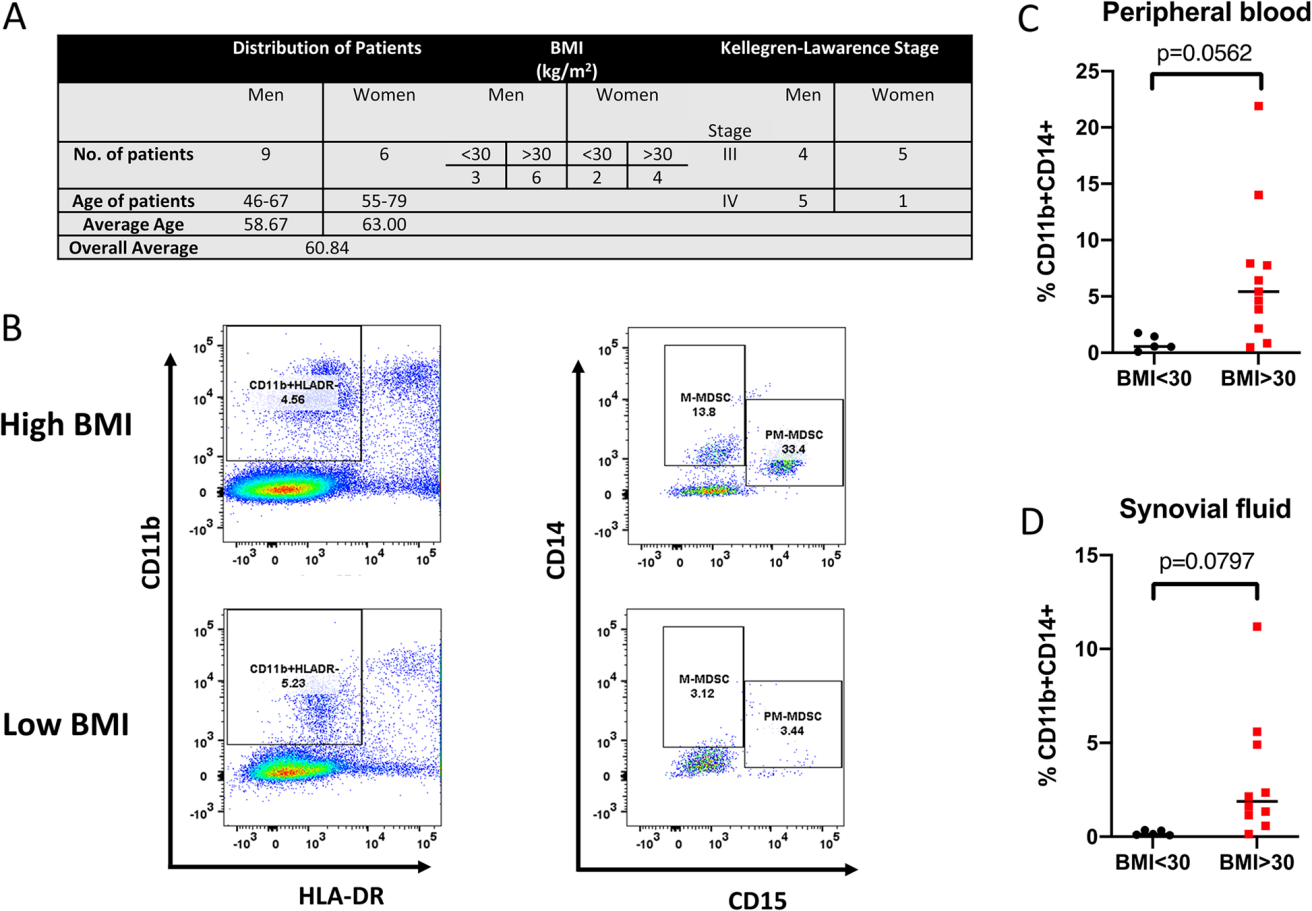
MDSCs are expanded in obese patients with post-traumatic osteoarthritis. **A** Demographic of the patient population. **B** Gating strategy used to define the M-MDSC subpopulation (CD11b^+^ HLADR^−^ CD14^+^ CD15^−^ cells). The M-MDSC population is expanded in **C** peripheral blood and **D** synovial fluid of obese (*BMI* > 30; *N* = 10) compared to non-obese (*BMI* < 30; *N* = 5) patients diagnosed with post-traumatic osteoarthritis. Two-tailed unpaired Student *t*-test

MDSC populations in 15 OA patients who were obese (>30 kg/m^2^; *n* = 10) or non-obese (<29.9 kg/m^2^; *n* = 5) were determined by flow cytometry. A summary of patient demographics with Kellgren-Lawrence stage of OA disease is presented (Fig. 5A) where all patients exhibited later-stage disease. The gating strategy for PMN-MDSC(HLA-DR^−/low^CD11b^+^CD14^−^ CD15^+^) and M-MDSC(HLA-DR^−/low^ CD11b^+^CD14^+^CD15^−^) quantitation in peripheral blood and synovial fluid of OA patients is presented in Fig. 5B. We observed that M-MDSC levels (Fig. 5C) in obese OA patients (median 6.12%, range 0.84–21.9%) were increased compared with non-obese OA patients (median 0.882%, range 0.54–1.77%); however, this did not quite reach significance. In synovial fluid, there was a trend towards M-MDSC cell expansion in obese OA patients compared with non-obese OA patients (Fig. 5D). Although PMN-MDSC populations appear to be reduced in Fig. 5B, we did not observe any significant differences when all patients were compared (data not presented).

## Discussion

Numerous studies have focused on the interactions between cartilage, bone, and the synovium in OA. Subchondral bone and hematopoietic stem cells play crucial roles in the initiation and progression of cartilage degeneration and OA pathology through bone remodeling where osteoclast activity is the instigating element [36–39]. Increased bone remodeling ultimately results in increased subchondral bone volume and density (sclerosis), which are both features of late-stage OA disease identifiable on X-ray film [40]. However, earlier boney changes in the subchondral bone are detected on magnetic resonance images (MRI) and described as bone marrow lesions (BMLs) which precede joint degeneration and thus present a possible predictive candidate for OA pathology [39]. In the present study, we show that there is an increase in osteoclast formation in DMM-induced OA in mice in the subchondral bone, where there were more osteoclasts found in obese mice than non-obese DMM OA controls. These data are consistent with others who have shown increased numbers of osteoclasts following surgically induced OA in mice in subchondral bone [41–44]. Similarly, others have shown that HFD results in increased OA disease pathology [45, 46]; however, this is the only study to date that indicates an increased level of osteoclasts in subchondral bone during obesity.

Since their initial description in cancer patients nearly two decades ago, MDSCs have been linked to several different types of inflammatory-associated pathologic conditions. In the present study, we observed that MDSCs are expanded in mice and humans during obesity with OA. MDSCs were expanded in peripheral blood of OA patient subjects relative to body mass index and in mice fed a high-fat diet (HFD) compared to mice fed a low-fat diet (LFD). Although other myeloid cells share the CD11b^+^Ly6C^+^ phenotype, we demonstrated that this population also suppressed T-cell subsets from animals fed an HFD, consistent with their immunosuppressive activity. However, additional in vitro experiments are necessary to definitively characterize this population [47]. M-MDSCs from obese mice had a greater capacity to form osteoclasts in culture with increased subchondral bone osteoclast number. These data are similar to a recent report that indicated that macrophages were a major component of obesity-associated OA and depletion of macrophage using clodronate-loaded liposomes decreased obesity-associated OA in mice [45]. In this study, we show that M-MDSCs were expanded in mice and humans with enhanced osteoclastogenic capacity and increased osteoclasts found in the subchondral bone area following histopathological examination. Similar to Sun et al., we tried a depletion strategy to address the role of a specific cell population using an anti-GR-1 antibody to deplete MDSC populations. However, this strategy failed to deplete MDSCs as determined by flow cytometry without seemingly any effect on OA histopathology. Other studies have reported issues with this antibody for MDSC depletion [48]. Nevertheless, the positive association of M-MDSC expansion and ability to differentiate into osteoclasts more efficiently from obese mice suggests a plausible role of M-MDSCs to migrate to the subchondral bone area to participate in the bone uncoupling process.

In addition, we have shown that obesity increased osteoclast number and number of nuclei within the osteoclasts. Indeed, it is well established that an increased number of nuclei in osteoclasts positively correlates with osteoclastic activity [49]. These data, coupled with human data where M-MDSCs are expanded in peripheral blood from OA patient subjects as well as supportive data that they appear to be expanded in the synovial fluid of obese OA patients, suggests that they could likely contribute towards OA pathology through increased osteoclastic activity and subchondral bone changes. However, additional studies would be needed to verify these data and provide more mechanistic data.

Over a decade ago, investigators noted that diet-induced obesity caused alterations in bone remodeling [50, 51]. Obesity has been shown to enhance MDSC expansion and obesity-related metabolic factors [18]. More recently, a number of reports have demonstrated that MDSCs derived from either BM or spleen can differentiate into osteoclasts [10, 15–17]. It should be noted that although there are several murine studies showing increased osteoclastogenesis potential in diet-induced obesity, few mechanistic studies have been conducted. These studies are mostly descriptive in nature showing changes in inflammatory cytokines that are usually associated with adiposity and osteoclastogenesis, including TNF-α, IL-1β, IL-6, and RANKL, that would be altered in the bone marrow microenvironment [51, 52]. In a recent study from our group, we utilized a focused transcriptomic platform to interrogate differential gene expression M-MDSC differentiation into osteoclasts. We observed several mRNAs were increased in M-MDSCs derived from HFD mice within the RANKL-stimulated signaling pathway, including well-known osteoclastogenic signaling intermediates or definitive marker genes of osteoclast differentiation including *Traf6* (tumor necrosis factor receptor (TNFR)-associated factor 6), *Csf1r* (colony-stimulating factor 1 receptor), *Jun* (transcription factor AP-1), *Pparg* (peroxisome proliferator-activated receptor gamma), and *Calcr* (calcitonin receptor) with the most significant increase in *Calcr* mRNA expression. Interestingly, *Oscar* (osteoclast-associated immunoglobulin-like receptor) was increased in HFD-derived M-MDSCs indicating both canonical and non-canonical osteoclastogenic pathways are activated during obesity-induced osteoclastogenesis. The present study extends these concepts into murine OA models and potentially humans. In the present study, we did observe that HFD itself increased M-MDSC expansion as well as the ability of DMM trauma to dramatically increase both expansion and differentiation capacity of MDSC populations derived from BM in metabolically challenged mice. In addition, data from both peripheral blood and synovial fluid from OA patients with effusion suggests that M-MDSCs are expanded systemically and locally in obese subjects.

## Conclusion

Consistent with the critical role of osteoclast-mediated bone resorption in OA pathology, treatment of OA with many anti-resorptive therapeutics shows efficacy in the clinical management of OA. Indeed, bisphosphonates, including zoledronate, cathepsin K inhibitors, and other anti-resorptive agents, have shown promise in experimental OA models [53–55]. These studies highlight the prospective that subchondral bone acts in concert with other tissues surrounding the joint and the early remodeling events, induced by osteoclasts, may offer a therapeutic window to delay or reverse the temporal sequence of events that occur during OA pathology. Our results demonstrate that MDSCs expand in the peripheral blood of OA patients and the bone marrow of OA mice and are associated with bone destruction, in particular in the context of obesity. Thus, M-MDSCs represent a potential new source of osteoclast precursors that contribute to bone destruction in OA which has immediate therapeutic target implications.

## Data Availability

The datasets used and/or analysed during the current study are available from the corresponding author on reasonable request.

## Abbreviations

PTOA: Post-traumatic osteoarthritis
OA: Osteoarthritis
DAMPs: Damage-associated molecular patterns
IL: Interleukin
TNF: Tumor necrosis factor
CD4: Cluster of differentiation 4
C3a: C3 activator fragment
MDSC: Myeloid-derived suppressor cell
PMN-MDSC: Polymorphonuclear myeloid-derived suppressor cell
M-MDSC: Monocytic myeloid-derived suppressor cell
LFD: Low-fat diet
HFD: High-fat diet
DMM: Destabilized medial meniscus
MFC: Medial femoral condyle
LFC: Lateral femoral condyle
LTP: Lateral tibial plateau
MTP: Medial tibial plateau
DIO: Diet-induced obesity
*BMI*: Body mass index
HLA: Human leukocyte antigens
DR: Antigen D related
PE: Phycoerythrin
APC: Allophycocyanin
FITC: Fluorescein isothiocyanate
